# Nuclear Receptor Small Heterodimer Partner in Apoptosis Signaling and Liver Cancer

**DOI:** 10.3390/cancers3010198

**Published:** 2011-01-05

**Authors:** Yuxia Zhang, Li Wang

**Affiliations:** Departments of Medicine and Oncological Sciences, Huntsman Cancer Institute, University of Utah School of Medicine, Salt Lake City, UT 84132, USA

**Keywords:** nuclear receptors, small heterodimer partner, apoptosis, cell cycle, liver cancer

## Abstract

Small heterodimer partner (*SHP*, NR0B2) is a unique orphan nuclear receptor that contains the dimerization and a putative ligand-binding domain, but lacks the conserved DNA binding domain. SHP exerts its physiological function as an inhibitor of gene transcription through physical interaction with multiple nuclear receptors and transcriptional factors. SHP is a critical transcriptional regulator affecting diverse biological functions, including bile acid, cholesterol and lipid metabolism, glucose and energy homeostasis, and reproductive biology. Recently, we and others have demonstrated that SHP is an epigenetically regulated transcriptional repressor that suppresses the development of liver cancer. In this review, we summarize recent major findings regarding the role of SHP in cell proliferation, apoptosis, and DNA methylation, and discuss recent progress in understanding the function of SHP as a tumor suppressor in the development of liver cancer. Future study will be focused on identifying SHP associated novel prooncogenes and anti-oncogenes in liver cancer progression and applying the knowledge gained on SHP in liver cancer prevention, diagnosis and treatment.

## 1. Introduction

Hepatocellular carcinoma (HCC) is one of the most common malignancies worldwide [1]. Chronic viral hepatitis associated liver cirrhosis, non-alcoholic steatohepatitis (NASH), ethanol consumption, hereditary diseases (α_1_ antitrypsin deficiency, hemochromatosis), and exposure to hepatotoxins (aflatoxin) represent the major risk factors for HCC development [2]. Apoptosis is a distinct form of cell death characterized by organized nuclear and cellular fragmentation that occurs in all organs and tissues. As in all other sites, a certain degree of hepatocyte apoptosis is characteristic of a healthy liver [3]. Indeed, in recent years it has become clear that development and progression of HCC are associated in particular with both defective apoptosis and increased cell proliferation. Specifically, tumor cells often show alterations in genes regulating the apoptotic machinery. However, the precise molecular mechanisms of apoptosis regulation involved in hepatocarcinogenesis are still poorly understood.

Nuclear receptors (NRs) are a unique family of transcription factors (TFs) that are being recognized as key regulators of multiple functions in almost all aspects of mammalian development, metabolism and physiology [4]. In recent years, accumulating evidence suggests that dysfunction of NR signaling leads to a wide spectrum of proliferative, reproductive, and metabolic diseases, including obesity, diabetes, and cancers. Small heterodimer partner (*SHP*, NR0B2) is a unique NR distinct from other conventional NRs in both its structure and function [5]. The presence of a putative ligand-binding domain (LBD) classifies SHP as a member of the NR family, although the endogenous ligand has not been identified. Importantly, the absence of the classical DNA binding domain (DBD) makes SHP an atypical NR, exerting its regulatory function through protein-protein interactions with other NRs and TFs. Since its discovery in 1996, SHP has been identified as a key transcriptional regulator affecting diverse biological functions, including bile acid, cholesterol and lipid metabolism, glucose and energy homeostasis, and reproductive biology [6]. Recently, we and others have found that SHP is an epigenetically regulated transcriptional repressor that suppresses the development of liver cancer in both humans and mice [7-9]. In this review, we will summarize major findings in the field regarding the role of SHP in cell proliferation, apoptosis, and DNA methylation, and discuss recent progress in understanding the function of SHP as a tumor suppressor in the development of liver cancer.

## 2. SHP Structure and Expression

SHP gene resides on chromosome 1 at 1p36.1 in humans and chromosome 4 and 5 in mouse and rat, respectively. The genomic structure of SHP consists of two exons, with a single intron spanning approximately 1.8 kilobases in humans and 1.2 kilobases in mouse [10]. The SHP gene is detected in a variety of tissues. In the mouse, SHP is predominantly expressed in the gallbladder and liver, and at lower levels in the brainstem, cerebellum, adrenal, pancreas, stomach, duodenum, jejunum, ileum, colon, kidney, ovary, testis, and heart [11]. In humans, SHP mRNA has been detected in the liver, heart, pancreas, kidney, spleen, small intestine, adrenal gland and stomach [10,12,13].

Accumulating evidence shows that many NRs and TFs can target the SHP promoter and regulate SHP gene expression. They include steroidogenic factor-1 (SF-1), liver receptor homologue-1 (LRH-1), farsenoid X receptor (FXR), c-jun, hepatocyte nuclear factor 4α (HNF4α), estrogen receptor-related receptor γ (ERRγ), E2A gene products (E47, E12 and E2/5), liver X receptor α (LXRα), estrogen receptor α (ERα), sterol regulatory element binding protein 1c (SREBP-1c), adaptor protein (AP1), pregnane X receptor (PXR), the core circadian component CLOCK-BMAL1, peroxisome proliferator-activated receptor γ (PPARγ), and upstream stimulatory factor-1 (USF-1) [12,14-26].

SHP is an unusual orphan member of the NR family. A classical NR contains five major functional domains: the N-terminal ligand-independent transactivation domain, the DBD, hinge region, the C-terminal LBD, and the ligand-dependent transactivation domain. However, SHP contains a putative LBD domain, but lacks the conserved DBD [5]. SHP interacts with other NRs or TFs through two functional LXXLL-related motifs (also called NR-boxes), which are located in the putative N-terminal helix 1 of the LBD and in the C-terminal region of helix 5. SHP generally represses the transcriptional activities of its target genes. SHP has been shown to bind directly to a variety of NRs, including SF-1, LRH-1, HNF4, HNF6, ERRs, LXRs, PPARs, GR, ERs, TRβ, RARα, FXR, PXR, CAR, AR, NGFI-B (Nur77) and RXRs (the common heterodimerization partner for many NRs). SHP also interacts with several TFs and co-regulators such as ARNT, BETA2/NeuroD, C-jun, Jun D, C/EBPα, Foxo1, HNF3, NF-κB, Smad3, P53, BAF155, BAF57, Brm, EID1, G9a, HDAC1, HDAC3, HDAC5, HDAC6, mSinA, SMRT, SIRT1, CBP/p300, and SRC-1. To date, three distinct transcriptional repression mechanisms for SHP have been identified. SHP represses NR or TF mediated transactivation by competition for coactivator binding to NR, recruitment of SHP-associated corepressors, and inhibition of DNA binding. SHP can utilize these three inhibitory steps alternatively or sequentially in a cell type and target gene specific manner. Although generally a repressor of gene transcription, SHP has also been found to activate NF-κB and up-regulate the transcriptional activity of PPAR [13,27]. SHP modulates gene transactivation or suppression by recruiting mSin3A/HDAC corepressors, class III histone deacetylase SIRT1, G9a methyltransferase, and Swi/Snf–Brm chromatin remodeling complex [28-31].

## 3. SHP and Apoptosis

Apoptosis is a distinct form of cell death characterized by organized nuclear and cellular fragmentation. It can be executed via two fundamental pathways: (i) the intrinsic mitochondrion-dependent pathway; or (ii) the death receptor (DR)-mediated extrinsic mitochondrion-independent pathway [32]. The intrinsic pathway is triggered by a variety of stressors and is mediated through the release of pro-apoptotic proteins that activate caspase enzymes from the mitochondria. The permeability of the mitochondrial membrane is tightly controlled by the balance of activity between pro- and anti-apoptotic members of the Bcl-2 superfamily proteins. Upon membrane permeabilization, cytochrome c and the pro-apoptotic protein smac/Diablo are able to translocate from the intermembrane space of the mitochondria into the cytosol. Cytochrome c then interacts with the adapter protein Apaf-1, dATP, and caspase-9 to subsequently activate caspase-3, leading to cell death [33]. The extrinsic pathway begins outside the cell through the activation of specific proapoptotic receptors on the cell surface. It is mediated through binding of specific molecules known as pro-apoptotic ligands to their death receptors, including CD95 (Fas/APO-1), TNF-R1, and the TRAIL receptors DR4 and DR5 [34]. Ligand binding results in direct cleavage and activation of procaspases-8 to further activate executor caspase-3, 6, and/or 7, thereby converging on the intrinsic pathway.

SHP is a pleiotropic regulator, influencing expression of multiple target genes involved in diverse biological processes, including regulation of metabolic pathways, stress and inflammatory response, detoxification, cellular adhesion and differentiation, and cell cycle control [35]. Recently, we and others have found that SHP contributes to regulation of apoptosis pathways.

### 3.1. SHP Functions in Apoptosis

**Figure 1.**
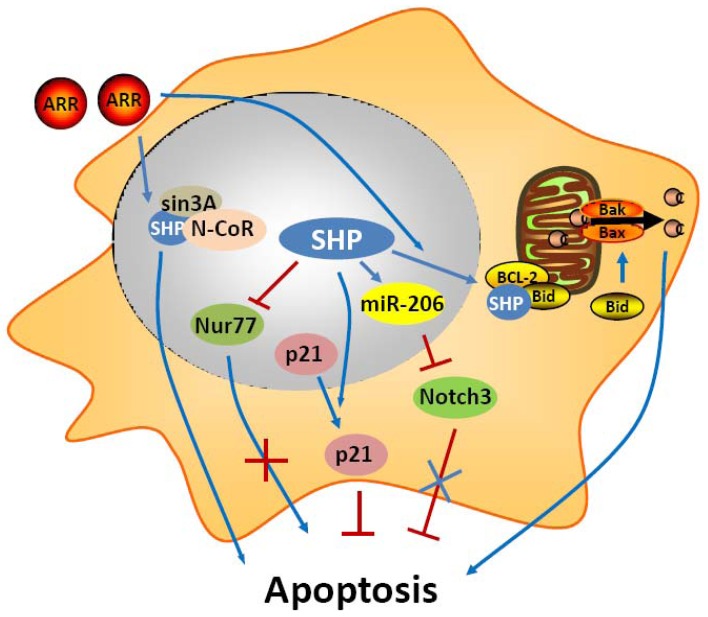
Role of SHP in apoptosis signaling. SHP is proposed to have both inhibitory and stimulatory effects on apoptosis (depending on cell types). SHP represses apoptosis through inhibiting the transcription of Nur77 and inducing the cytoplasmic sequestration of p21WAF1. SHP activates apoptosis by translocating to mitochondria, binding to the anti-apoptotic protein Bcl-2, and disrupting Bcl-2/Bid interaction to cause cytochrome c release. SHP also activates apoptosis through regulating miR-206 expression to block the anti-apoptotic activity of Notch3. The adamantyl-substituted retinoid-related (ARR) compounds AHPN and 3-Cl-AHPC bind directly to SHP, which promotes the formation of a corepressor complex containing Sin3A, nuclear receptor co-repressor (*N*-CoR) to activate apoptosis. ARRs also increase SHP protein stability by preventing its degradation and enhance SHP mitochondrial targeting. Activation pathways are shown as blue arrows, and inhibitory pathways are shown as red lines.

In an early study, SHP was shown to have a negative crosstalk with Nur77 during anti-Fas antibody (CH11) mediated apoptosis of hepatic cells [36] (Figure 1). Nur77 is an orphan nuclear receptor that has been identified in apoptosis of a variety of cell types, including hepatocytes. In prostate and lung cancer cells, upon apoptotic stimuli, Nur77 translocates from the nucleus to the cytoplasm where it targets mitochondria to induce cytochrome c [37,38]. In these cells, the apoptotic effect of Nur77 does not require its transcriptional activity or DNA binding, whereas the interaction of Nur77 with the Bcl-2 apoptotic machinery converts Bcl-2 from a protector to a killer [39]. In contrast, in liver cells, the transactivation function of Nur77 plays a critical role in the regulation of the Fas/FasL-induced apoptotic pathway. SHP was shown to bind the coactivator CBP, thereby releasing the coactivator from Nur77, resulting in repression of the transcriptional function of Nur77. Consistent with this, expression of SHP decreased, whereas antisense SHP enhanced, the transcriptional activity of Nur77 in human liver cancer HepG2 cells. Further, SHP was not detected in the interferon γ (IFNγ)/CH11-sensitive liver cancer SNU354 cells, whereas it was significantly expressed in the IFNγ/CH11-resistant HepG2 cells. Moreover, SNU354 cells stably expressing SHP became resistant to IFNγ/CH11-induced apoptosis. From these observations, SHP has been proposed to play a protective role in Nur77 mediated apoptosis in liver.

Another recent study shows that SHP expression was increased during monocytic differentiaton through c-Jun and p65 mediated transcriptional activation of the SHP promoter [40]. Interestingly, SHP protected differentiated monocytic cells from etoposide-induced cellular apoptosis through the induction and cytoplasmic sequestration of p21WAF1. Cytoplasmic p21 has been demonstrated to exert biological effects distinct from those of nuclear p21 [41]. p21 functions as a cell cycle brake in the nucleus and as an inhibitor of apoptosis in the cytoplasm. This study indicates that SHP participates in the monocytic differentiation program and contributes to the acquisition of resistance to apoptosis by inducing cytoplasmic localization of p21.

In contrast to these two studies about the anti-apoptotic function of SHP, we and others have demonstrated that SHP is an active component of apoptosis signaling [42-47]. The adamantyl-substituted retinoid-related (ARR) compounds 6-[3-(1-adamantyl)-4-hydroxyphenyl]-2-naphthalenecarboxylic acid (CD437/AHPN) and 4-[3-(1-adamantyl)-4-hydroxyphenyl]-3-chlorocinnamic acid (3-Cl-AHPC) are effective inducers of apoptosis of malignant cells both *in vitro* and *in vivo*. Although numerous potential mechanisms have been proposed for how these compounds exert this effect, the precise mechanism by which ARR induces apoptosis remains unclear. An exciting recent observation is that AHPN and 3-Cl-AHPC are SHP ligands and induce apoptosis in human leukemia cell lines HL-60R and KG-1, and the breast carcinoma cell line MDA-MB-468 through their binding to SHP [43]. The binding of ARRs to SHP consequently promotes the formation of a corepressor complex containing Sin3A, nuclear receptor co-repressor (N-CoR), histone deacetylase 4, and histone shock protein (HSP90). This protein complex in turn binds to the c-myc promoter and inhibits its transcription. Knockout of SHP and knockdown of Sin3A compromise the proapoptotic activity, indicating that formation of the SHP-Sin3A complex is essential for the ability of ARRs to induce apoptosis. In addition, the correlation between ARRs binding to SHP and the induction of apoptosis strongly suggests that SHP functions as the receptor through which the ARRs exert apoptotic activity. However, the mechanism by which binding of ARRs to SHP modulates the SHP-Sin3A complex remains to be delineated. Nevertheless, the loss of SHP or Sin3A expression had little effect on ARR inhibition of cellular proliferation, suggesting that ARR mediated growth inhibition and apoptosis seem to involve separate pathways [48]. Continued study showed that induction of c-Fos and c-Jun expression as well as NF-kappaB activation by ARRs were SHP-dependent and may be essential for ARR mediated apoptosis [45]. A more recent *in vivo* and *in vitro* study found that the human acute myelogenous leukemia cell line FFMA-AML and TF (v-SRC) cells displayed resistance to standard retinoid (including trans-retinoic acid, 9-cis-retinoic acid, and the synthetic retinoid TTNPB) induced apoptosis but showed sensitivity to 3-Cl-AHPC and AHPN mediated apoptosis. Detailed experiment showed the decreased expression of the antiapoptotic proteins (cellular inhibitor of apoptosis 1 and X-linked inhibitor of apoptosis protein) and phospho-Bad, and the activation of the NF-kappaB canonical pathway [46]. Interestingly, knockdown of SHP in these AML cells blocked 3-Cl-AHPC and AHPN mediated induction of apoptosis. Overall, these results provided evidence that SHP has important functions in ARR induced apoptosis.

Recently, we used a series of *in vivo* and *in vitro* experimental approaches to demonstrate that SHP is a critical component of mitochondrial apoptotic signaling and mediates the proapoptotic effects of AHPN [47]. We found that SHP overexpression in mouse HCC Hepa-1 cell was sufficient to induce apoptosis and also enhanced greatly the apoptotic effects of TNF-α and AHPN [47]. We also observed that SHP activated apoptotic signaling in mouse hepatocytes, as evidenced by the decreased number of apoptotic hepatocytes in *SHP*^-/-^ livers, and the increased apoptosis in hepatocyte-specific SHP-transgenic (STG) mice. The STG mice were also hypersensitive to Fas-induced hepatocyte apoptosis. Interestingly, both AHPN and AHPC were able to activate SHP gene transcription via LRH1 and induce apoptosis in human liver cancer Huh7 cells. Of particular interest in these studies are the observations that a competition exists between AHPN and HNF4α to traffic SHP between mitochondria and the nucleus, and that SHP interacts with Bcl-2 in mitochondria to induce cytochrome *c* release. Bcl-2 normally functions as an anti-apoptotic protein by inhibiting the effect of pro-apoptotic proteins. Upon mitochondrial localization, SHP interacts with Bcl-2 and disrupts the Bcl-2/Bid complex, resulting in activation of apoptosis. We are currently in the process of further delineation of endogenous signaling molecules that are involved in SHP mitochondrial targeting. Interestingly, we also observed a feedback loop in which Bcl-2 decreased SHP protein stability (Zhang, Y.X. and Wang, L., manuscript in preparation). Perhaps the most exciting result from these studies is the discovery that induction of SHP by oral administration of AHPN serves as a potent inhibitor of *in vivo* implanted peritoneal pancreatic tumor growth. Therefore, our findings suggest the intriguing possibility of manipulation of SHP in the treatment of hepatic and other gastrointestinal cancers.

MicroRNAs (miRNAs, miRs) are recently discovered small 21-23 nts noncoding RNAs that repress the expression of their target genes primarily by a post-transcriptional mechanism. MiRNAs participate in the regulation of many cellular processes including apoptosis, and alteration of their expression is frequently observed in cancers. Recently, we identified a novel “dual inhibitory” cascade regulation governing the activation of miR-206 gene transcription by SHP, which involves ERRγ, YY1, and AP1 (c-Jun and c-Fos) [49]. A promising observation is that expression of miR-206 markedly induces apoptotic cell death and blocks the anti-apoptotic activity of Notch3. Thus, SHP also activates apoptosis through the regulation of miR-206 expression and function.

### 3.2. FXR Functions in Apoptosis

Nuclear receptor FXR, a SHP transcriptional activator, is best known for regulating the homeostasis of cholesterol and bile acids, but its role as a mediator of apoptosis has been increasingly recognized in various types of cells [50-54]. The earlier studies have described the use of FXR agonists as enhancers of apoptosis in ovarian cancer cells and in vascular smooth muscle cells [50,53]. However, it remains unknown how FXR activation induces apoptosis. Based on our studies, a possible explanation is that FXR activation induces SHP gene transcription which in turn induces apoptosis. One study demonstrates that exposure of hepatic stellate cells (HSCs) to 6-ethyl chenodeoxycholic acid (6-ECDCA), a FXR ligand, increases SHP expression. This then causes an inhibition of Jun D binding to the consensus element in the tissue metalloproteinase inhibitor (TIMP-1) promoter, resulting in TIMP-1 transcriptional inhibition, and also enhances the sensitivity of HSCs to proapoptogenic stimuli [51]. This study established that FXR ligands may be beneficial in treatment of liver fibrosis. Swales *et al.* reported that FXR is present in human breast cancer MCF-7 and MDA-MB-468 cells, and that activation of FXR by high concentrations of its ligands activates SHP gene transcription and induces cell apoptosis [52]. Thus, this study suggests the possibility of manipulating FXR and SHP in the treatment of breast cancer. FXR is abundantly expressed in the liver and the lower digestive tract. In the human colon, the expression of FXR has been reported to progressively decrease in the sequence normal mucosa – adenoma – adenocarcinoma [55]. In addition, reactivation of FXR in the intestine and colon cancer cells results in induction of apoptosis [54]. Therefore, from a therapeutic standpoint, the above results provided evidence that strategies aimed at inducing FXR/SHP expression to activate apoptosis might be useful in treating liver fibrosis, breast and liver cancer.

In contrast, FXR has been demonstrated to play a protective role in inhibiting liver cell apoptosis induced by nutritional withdrawal [56]. FXR activation by CDCA (50 μM/L) and GW4064 (2 μM/L) suppresses serum deprivation-induced apoptosis in human liver cancer HepG2 cells by activating the ERK1/2 MAPK pathway. Moreover, *FXR*^-/-^ mice showed enhanced liver cell apoptosis induced by starvation, suggesting a protective role of FXR in maintaining normal liver physiology. This study is consistent with the observation that guggulsterone, a potent antagonist of FXR, induced apoptosis in a Barrett's esophagus-derived cell line [57]. Because guggulsterone also acts as a steroid receptor ligand, this study cannot exclude the possibility that apoptosis is induced by other mechanisms not involving FXR. At present, it is unclear whether those incongruities of SHP and FXR functions in apoptosis could potentially reflect differences in the surrounding microenvironment, tissue specific effects, or different technologies employed in the studies.

## 4. SHP and Liver Cancer

NRs have been implicated in the initiation and progression of a wide variety of cancers. Prostate cancer has been linked to the androgen receptor, bone cancer to the nor-1 receptor, colon cancer to PPARγ, breast cancer to estrogen receptors, and leukemias to nur77. Due to their important physiologic functions in cancers, NRs and their regulated critical genes are emerging targets for molecular diagnosis and cancer therapeutics. For instance, by competing with endogenous estrogen, anti-estrogenic drugs have been successfully applied in the treatment of breast cancers that have excess estrogen receptor activity. Our recent studies have demonstrated that SHP has potent tumor suppressive functions in HCC via inhibition of cellular growth and activation of apoptosis [7,8,47].

### 4.1. SHP and Cell Proliferation in HCC

**Figure 2.**
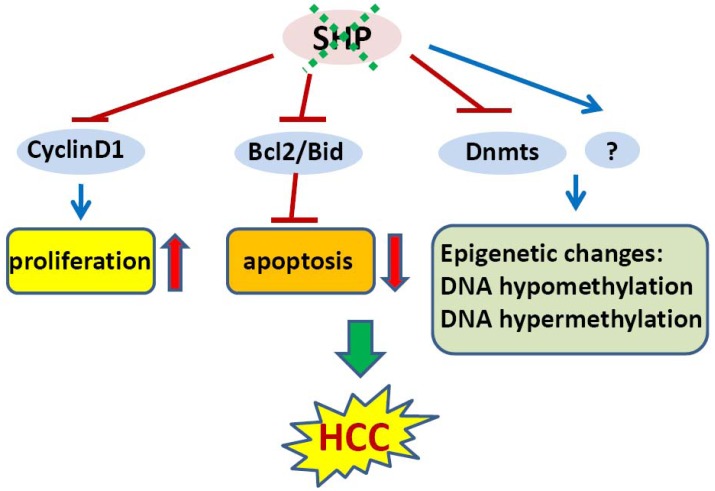
Role of SHP in the development of HCC. The loss of SHP increases hepatocyte proliferation (though cyclinD1), decreases apoptosis (though Bcl2), and results in genome wide epigenetic changes (through Dnmts), including DNA hypomethylation and the hypermethylation of tumor suppressor genes. These events coordinately promote the development of HCC.

In an initial study, we showed that SHP negatively regulates cellular growth and the deletion of SHP results in enhanced hepatocyte proliferation [8] (Figure 2). *SHP*^-/-^ mice developed spontaneous HCC at 12 to 15 months of age, which is associated with massive hepatocyte proliferation and increased cyclin D1 expression. Conversely, cell proliferation is completely reversed in hepatocyte specific *SHP* transgenic mice. Consistent with the *in vivo* observation, enhanced proliferation and increased cyclin D1 expression were observed in *SHP*^-/-^ embryonic fibroblasts. SHP was further shown to repress cyclin D1 gene transcription through LRH-1. We also discovered that the immortal *SHP*^-/-^ fibroblasts displayed characteristics of malignant transformed cells and formed tumors in nude mice. Of particular note, this study revealed a unique role for SHP in mediating cell growth through repression of cyclin D1, thereby providing a novel molecular link between SHP and liver cancer. Interestingly, Smith *et al.* demonstrated recently that sodium taurocholate (NaTC) decreases intestinal tumor formation in APC ^Min/+^ mice. This effect is mediated through the activation of FXR and suppression of cyclin D1 by SHP, resulting in reduced epithelial cell proliferation [58].

### 4.2. Epigenetic Silencing of SHP in HCC

The expression of SHP is markedly diminished in human HCC compared with the normal surrounding liver tissue because of SHP promoter hypermethylation [7]. We further demonstrated that methylation occurred more frequently in the six sparsely distributed CpG orphans in the SHP proximal promoter than in the exonic CpG island of the SHP gene. Of particular interest, we discovered that methylation of the six CpGs *in vitro* blocked LRH-1 binding and transactivation of the SHP promoter by recruiting methyl-CpG binding proteins and co-repressors. In contrast, the normal surrounding tissues had fewer methylated CpGs in this region. Therefore, methylation of the six CpGs appeared to be critical for maintaining normal SHP promoter activity and played a major role in silencing SHP during HCC progression. Consistent with the tumor suppressor functions of SHP, overexpression of SHP inhibited human HCC foci formation, arrested HCC tumor growth in xenografted nude mice, and increased the sensitivity of HCC cells to apoptotic stimuli. Overall, this study suggests that CpG hypermethylation is an important mechanism in SHP gene silencing and the loss of SHP expression might be a critical epigenetic event in the development of liver cancer.

In line with our observations, a more recent study revealed that SHP is a good prognostic factor in HCC [9]. Notably, by using genomic approaches, this study identified several cell cycle genes that are regulated by SHP in liver cancer, including CDK4, MCM5, EXOCS1, CCNB1, BUB3, and BCL2L2. The poorer survival of liver cancer patients with low expression of SHP might be due to higher proliferation of cancer cells.

### 4.3. SHP and Epigenetic DNA Methylation in HCC

Global changes in DNA methylation status as well as changes in methylation patterns of individual genes are characteristic properties of a wide variety of tumor cells. Both DNA hypomethylation and hypermethylation are associated with cancer [59]. Hypomethylation of specific proto-oncogenes and hypermethylation of tumor-suppressor genes contribute to a selective growth advantage of the cancer cells and may therefore represent one of the multiple steps leading to malignancy [60]. For instance, hypomethylation of proto-oncogenes such as c-fos, c-myc, Ha-ras and Ki-ras genes has been observed particularly in human liver cancer. Conversely, early hypermethylation changes appear to involve p15 (INK4B) and p16 (INK4A) in cirrhosis, and p16 (INK4A) in HCC [61].

In mammalian cells, DNA methylation occurs by adding a methyl group to the 5-carbon position of the cytosine residue in the CpG dinucleotide, which is catalyzed by the family of DNA methyltransferases including DNMT1, DNMT2, DNMT3A, DNMT3B, and DNMT3L. DNMT1 maintains DNA methylation during replication by copying the methylation pattern of the parent DNA strand onto the newly synthesized strand. DNMT2 only has weak DNA methylation ability *in vitro* and appears to be involved in methylation of RNA. DNMT3A and DNMT3B are responsible for *de novo* DNA methylation. DNMT3L is expressed during gametogenesis and establishes maternal genomic imprinting. Despite the important roles of DNMTs in liver cancer formation, how the expression of these genes is regulated remains poorly understood. Our recent study identified SHP as a transcriptional repressor of DNMT1 expression. This raises the question whether aberrant DNA methylation exists and contributes to the development of HCC in *SHP* deficient conditions. We combined a genome wide analysis of DNA methylation and gene expression to compare the differential methylation status of a comprehensive set of genes in normal liver vs. *SHP*^-/-^ liver. We found that promoter DNA methylation is aberrantly distributed in the *SHF*^-/-^ genome compared with normal liver genome (Zhang, Y.X. and Wang, L., manuscript in preparation). Therefore, the loss of SHP function appears to be correlated with epigenetic changes, which might contribute to the development of liver cancer.

## 5. Perspective

SHP is a unique member of the nuclear receptor superfamily in both its structure and function. The fact that SHP acts as a critical transcriptional coregulator in diverse metabolic processes provides a potential means to develop SHP targeted therapeutics for several metabolic diseases. Of particular interest is the observation that manipulation of SHP through the synthetic ligands ARRs induces apoptosis and inhibits tumor growth *in vivo*. This raises the possibility of developing synthetic agonists that activate SHP selectively as therapeutics for liver fibrosis and cancer. Major challenges for future research are to delineate the ARR binding pocket in the SHP molecule, characterize those motifs that are critical for binding, determine whether endogenous SHP ligands exist, and develop more efficacious and selective synthetic SHP ligands for pharmaceutical applications. The identification of SHP in the mitochondria opens new doors for future investigation of the endogenous signaling molecules that initiate SHP mitochondrial targeting. SHP protein stability was increased in the AHPN induced apoptosis pathway, and it would be interesting to determine whether the stability of SHP is crucial in regulating other pathways such as lipid and glucose metabolism.

## 6. Conclusions

Studies by us and others provide strong evidence that SHP functions as a tumor suppressor in the development of liver cancer by inhibiting cell proliferation and inducing apoptosis. Of particular note, the loss of SHP results in genome wide aberrant DNA methylation, which may have implications for investigation and treatment of liver cancer. Although much work still remains to be completed, we are excited by the prospects of applying SHP as a new diagnostic, therapeutic and preventive agent for liver cancer. In this context, it is of particular interest to identify SHP associated novel pro-oncogenes and anti-oncogenes.
